# The Effect of Human–Horse Interactions on Equine Behaviour, Physiology, and Welfare: A Scoping Review

**DOI:** 10.3390/ani11102782

**Published:** 2021-09-24

**Authors:** Katherine Jennifer Kelly, Laurie Anne McDuffee, Kimberly Mears

**Affiliations:** 1 Interdisciplinary Studies, University of New Brunswick Saint John, Saint John, NB E2K 5E2, Canada; kj.kelly@unb.ca; 2 Health Management, Atlantic Veterinary College, Charlottetown, PE C1A 4P3, Canada; 3 Data and Research Services, Robertson Library, University of Prince Edward Island, Charlottetown, PE C1A 4P3, Canada; kmears@upei.ca

**Keywords:** human–horse interactions, human–horse relationship, human–horse bond, animal interaction, scoping review

## Abstract

**Simple Summary:**

Human–horse interactions (HHIs) are an important aspect of society, especially in the equine industry. HHIs are diverse and can be focused on horses as an economic means, pleasure, or companionship for humans. As a result, the welfare of horses during these interactions, including their mental and physical health, is an important consideration. Although the physical health of horses can be readily measured during equestrian activities, their mental health is more difficult to assess. This review was conducted to evaluate what is known about the horse’s mental state during common HHI in an attempt to better understand the welfare of the horse.

**Abstract:**

Human–horse interactions (HHIs) are diverse and prominent in the equine industry. Stakeholders have an invested interest in making sure that HHIs are humane. Assessment of equine welfare goes beyond physical health and includes assessment of the emotional state of the animal. HHIs can have a permanent effect on human–horse relationships, thereby influencing welfare. Therefore, an understanding of the horse’s affective state during HHIs is necessary. A scoping review was conducted to: (1) map current practices related to the measurement of HHIs; (2) explore the known effects of HHIs on horse behaviour and physiology; and (3) clarify the connection between HHIs and equine welfare. A total of 45 articles were included in this review. Studies that used both physiological and behavioural measures of equine response to human interactions accounted for 42% of the included studies. A further 31% exclusively used physiological measures and 27% used behavioural observation. Current evidence of equine welfare during HHIs is minimal and largely based on the absence of a negative affective state during imposed interactions. Broadening the scope of methods to evaluate a positive affective state and standardization of methodology to assess these states would improve the overall understanding of the horse’s welfare during HHIs.

## 1. Introduction

Horses were domesticated around 4000 B.C. and have long been valued for their important contributions towards human survival, development, and recreation [1]. Understanding the complex relationship between horses and humans has significant implications for safety, for both horse and human [2,3,4], as well as horse welfare [5,6]. 

The relationship between humans and animals is considered to be an evolving process, defined as a mutual perception that develops from mutual behaviour [7]. The relationship is developed from ongoing interactions which may have a positive or negative cumulative effect. The human–horse relationship, more specifically, is posited to benefit both species when developed through positive interactions and consistency [5,8]. There are many studies suggesting the benefit of human–horse interactions (HHIs) for humans, especially in regard to equine-assisted therapies (for reviews, please consult [9,10,11,12,13]); however, little is known about the effect of these interactions on the horses [6,14]. The lack of developmental standardization regarding the potential effect of therapy and other HHIs on animal welfare poses potential risks to both animals and humans [15]. 

In a systematic review on equine-assisted activities, O’Haire et al. [14] noted that no outcomes related to animal welfare were reported in identified primary studies. However, investigators argue that animal welfare is crucial to successful and ethical outcomes from human–animal interactions. As a result, the potential effect that these HHIs may have on equine welfare is unclear. This need for focused investigation on animal welfare relative to HHIs has been emphasized in the Five Domain Model [16]. This framework describes five critical areas relevant to animal welfare assessment and management: nutrition, environment, health, behaviour, and mental state. 

While assessment of many aspects of animal welfare can be straightforward, assessment of an animal’s mental state is more challenging. Because horses have been domesticated and can be readily trained and habituated to withstand aversive stimuli presented during HHIs [17], behaviour alone may not be an appropriate measure of mental state. Physiological measures obtained during HHIs can help to ascertain a horse’s mental state. Horses have been historically considered farm animals where they were used as working equids. HHIs have evolved to include horses as sport animals, companions, and more recently as therapy animals. The mental state of horses may be a particularly important consideration in horses used for therapeutic interventions with humans having mental health issues such as post-traumatic stress disorder (PTSD) [10] since entrainment theory is considered to occur during such HHIs [18]. Entrainment theory describes a process of mirroring in the interaction between independent mechanisms [19], such as between the physiology of the horse and human during therapy sessions. In other words, entrainment suggests that the functioning of a human’s psychophysiology may have an effect on the health of the animal. For example, it has been suggested that the emotional state of humans may have an impact on interactions between humans and horses [18,20], which may have implications for animal welfare [21]. A review of HHIs in various environments where horses are considered in these various roles and what they might reveal about the welfare of the horse is therefore warranted.

The human–horse relationship is well documented in the literature by three major reviews [5,18,22]. Hausberger et al. [5] explored the state of knowledge related to the interplay of several aspects of HHIs within a variety of equine related experiences and environments. This review highlighted the relevance of human management and care on equine interactions as a means to improve the human–horse relationship. Specifically, researchers emphasized the importance of positive interactions as a means to improve future interactions and improve human safety and equine welfare. In a more recent review, Clough et al. [22] focused on the nature of the human–horse relationship in horses used specifically for pleasure riding. Despite the extensive breadth of these two reviews, it remains largely unclear how interactions with humans affect the horse. Finally, Scopa et al. [18] highlighted the mechanisms that lead HHIs to become a relationship and the role of emotional transfer between the horse and human in the development of this bond. A clear understanding of HHIs and their effect on the horse perspective of humans has significant implications for equine welfare. The interconnection between equine learning, motivation, and stress mechanisms during interactions with humans are integral to horse welfare and management [23]. 

This paper aims to review current practice related to the measurement of HHIs and explore the known effects of these interactions on equine physiology and welfare. Previous research has suggested that a variety of tools are used to assess HHIs [5]. This study aims to systematically detail how the effects of these interactions are measured in the equine partner, the known effects of HHIs, and explore how HHIs affect the welfare of the horse. 

## 2. Methods and Materials

A scoping review was chosen to better understand the state of the literature on HHIs and its effect on the equine partner. Scoping reviews use structured methods for summarizing knowledge on a topic [24], particularly in topics that consist of diverse methods and disciplines [25]. Unlike other types of structured reviews, such as systematic reviews, scoping reviews allow for heterogeneity in methodological scope to identify gaps in knowledge areas [26]. 

The process for the current review was guided by Khalil et al.’s [27] evidence-based approach to conducting scoping reviews, using a methodology based on frameworks proposed by Arksey and O’Malley [28]; Levac, Colquhoun, and O’Brien [24]; and the Joanna Briggs Institute [29]. The development of the methodology consisted of the following five steps: (1) identify the research question(s); (2) identify relevant studies using a three-step literature search; (3) select studies using a team approach; (4) chart the data in tabular and narrative format; and (5) collate the results to identify implications for practice and research. This process, as it pertains to the current review, is described in the following five sections.

### 2.1. Identify the Research Question(s)

The current scoping review aimed to: (1) map current practice related to the measurement of HHIs; (2) explore the known effects of these interactions on equine behaviour and physiology; and (3) clarify the connection between HHIs and equine welfare. The following broad research questions were used to guide the present scoping review:

1. How are the effects of HHIs measured in the horse?
2. What are the known effects of HHIs on equine physiology?
3. How do HHIs affect the welfare of the horse?

This is the first review, to the authors’ knowledge, that has attempted to summarize the effect of HHIs specifically on equine physiology and welfare. However, the nature of the HHIs and human–horse relationship is well documented in previous reviews [5,22]. 

### 2.2. Identify Relevant Studies Using a Three-Step Literature Search 

A comprehensive three-step search strategy was developed by an experienced research librarian (KM) in consultation with the research team. The first step of the search strategy consisted of a search of two databases (PsycInfo and CAB Direct via EBSCOhost) to identify titles and abstracts of studies that examined the human–horse bond. The text words used in identified articles at this preliminary stage (e.g., in titles, abstracts, and keywords) were examined and used to identify additional keywords, subject headings, descriptors and related search terms. The second stage of the search strategy involved using the identified keywords to conduct a more comprehensive search of the literature. Searches for relevant articles were completed on 13 August 2019 in three electronic databases: PubMed, CAB Abstracts via the EBSCO host platform, and PsycInfo via the EBSCOhost platform. Updated searches in these same databases took place in September 2020 and June 2021. The syntax for the search strategy in each database is outlined in Table S1. 

The third step of the search strategy included a search for scientific evidence published in sources other than journals, such as peer-reviewed textbooks and publications from other sources, and evidence-based consensus expert opinion statements. The search consisted of a broad search on Google and several veterinary medicine and general databases (e.g., Open Grey) using the following keywords: “human horse bond” or “human horse relationship” or “human horse interaction”. A full list of the grey literature databases and corresponding keyword searches are available in Table S2. Sources were screened in Google according to titles until the point of saturation (i.e., after 2 pages passed in which a link was not opened).

### 2.3. Selection of Studies Using a Team Approach 

Citations from articles identified by the keyword searches were exported from their respective databases and imported into Rayyan QCRI, a free systematic review software that facilitates the organization and screening of articles [30].

#### 2.3.1. Eligibility Criteria 

A priori inclusion and exclusion criteria were established by the research team to guide the identification of relevant articles (see Table 1. In accordance with the research questions, articles were only included in the current review if their primary focus involved HHIs. This “bond” has also been referred to as an interaction [31], dyad [32], and relationship [5] in the literature. Articles were required to refer directly to the human–horse interaction/bond/dyad/relationship as a main focus to be considered for inclusion in the present review and specifically examine the effect of those interactions in the horse. Therefore, articles that exclusively examined the effect of the HHIs in humans (e.g., equine-assisted therapy) were not included. Studies were included in this study if the primary objective related to the measurement or description of encounters between horses and humans. Studies that investigated the effect of an intervention whereby the presence of the human is not considered (e.g., responses to object-based novel stimuli) were not included in the present review.

Articles were excluded if they did not focus specifically on the equine species (e.g., canine–human bond, etc). Articles were included if they reported primary research findings (i.e., reviews and editorials were not included) and were available as a full text in English. Finally, articles that focused on horses in developing countries, according to the United Nations’ Human Development Index (HDI) reports, were not included [33]. 

#### 2.3.2. Study Selection

Articles identified in the keyword searches underwent a careful process of selection to be included in the current scoping review. The selection of articles consisted of a screening of titles and abstracts, followed by a more in-depth screening of full-text articles. Duplicate articles were identified and removed by the lead author (KK). Two reviewers (KK and a research assistant) independently conducted the first level of title and abstract screening against the established eligibility criteria. A calibration test on 50 titles and abstracts was conducted to evaluate reviewer agreement in the screening process; this resulted in a kappa statistic of 0.716 (SE = 0.100, 86.79% agreement; measure of inter-rater agreement), which was considered sufficient for further independent screening [34]. Reviewers met to discuss any discrepancies, and a third reviewer (LM) resolved any outstanding conflicts.

The second stage of study selection consisted of the retrieval of full-text articles for included titles and abstracts, which were imported into Rayyan QCRI for further evaluation and data extraction. The same two reviewers independently screened full-text articles using the same process as the one described above. 

### 2.4. Chart the Data 

The two reviewers (KK and a research assistant) independently charted (i.e., extracted) data using a data extraction form developed by the research team using Google Drive (Table 2). We created columns and rows to describe the papers and their features, and piloted our spreadsheet for data extraction. Variables included: (1) information about the study; (2) methodological process; (3) description of HHIs; and (4) key findings of study. Inconsistencies in data extraction were reviewed and discussed among the members of the research team using an iterative process. Only findings related to the research questions were extracted for the purposes of this study; results that focused on the effect of HHIs on human participants were not considered. 

### 2.5. Collate the Results 

Through this scoping review, we aim to clarify any effects of HHIs on the physiology and welfare of the horse, including approaches to its measurement. Therefore, the results will be analyzed and presented in a narrative format, which will involve a qualitative thematic analysis of the results to illustrate key findings and themes. Thematic analysis is a flexible process of identifying, analyzing, and reporting patterns within a data set, providing a detailed and in-depth description of qualitative data [35]. Data analysis was completed by reading through studies, and then taking notes on first impressions. A second reading of the studies involved extracting information into a form (see Table 2) and creating sub-themes. Sub-themes were developed into major themes, as appropriate (see Table S3). Resulting themes provide an interpretation and synthesis of findings beyond the boundaries of individual studies to provide clarity on the effects of HHIs on the horse.

## 3. Results 

### 3.1. Selection of Included Articles 

**Figure 1:**
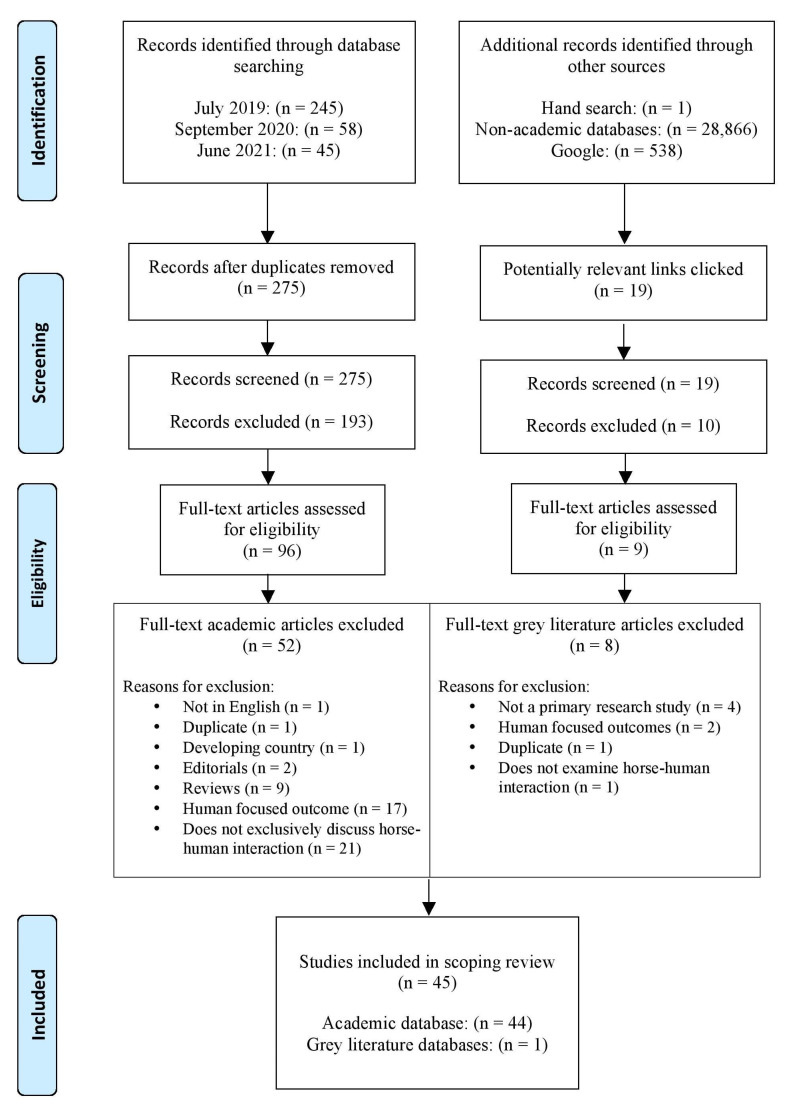
PRISMA flow chart [36].

A total of 348 articles was identified by the keyword searches across three databases (PsychInfo, CAB Abstracts, and PubMed). Specifically, 245 were identified in July 2019, 58 in September 2020, and 45 in June 2021. A further 28,866 sources were identified through a structured search of other literature, including Google and various veterinary medicine sources. After removal of duplicates, 275 academic articles underwent title and abstract screening, from which 193 were excluded. This resulted in 96 academic articles that underwent full-text screening, from which another 52 were excluded. A total of 19 potentially relevant sources were identified in the other literature search; 9 underwent full text evaluation, from which 8 were excluded. The reason that only 19 sources were evaluated from thousands identified is because the lack of advanced search tools in the other literature databases meant that many identified sources were not relevant. Detailed results from each of the other literature databases used in this study can be viewed in Table S2. The search strategy resulted in a total of 45 articles included in the current scoping review. Extracted data from articles are available in Table S4. A Preferred Reporting Items for Systematic Reviews and Meta-Analysis (PRISMA) flow chart outlines the search results according to each stage of the decision process in Figure 1 [36]. 

### 3.2. Article Characteristics 

#### 3.2.1. Study Populations 

Studies varied in the reporting of equine participants. A total of 1934 horses were used across all 45 studies, with a mean of 44 and median of 20, ranging from 3 to 339. One study, which observed three herds of undomesticated horses [37], did not report the number of equine participants. 

A total of 23 studies (51.1%) reported the breed of horses used in studies. Reported breeds were described as follows: various breeds (6); Dutch warmblood horses (2); standardbred (2); thoroughbred (2); Anglo-Arabian (1); Anglo-Arabs and Welsh ponies (1); Konik polski horses (1); Hanoverian Riding Horses (1); Małopolski horses (1); multiple (Swedish warm-blood horses, Andalusian) (1); ponies and a horse (1); ponies of unregistered mixed breed (1); Welsh mares (1); and working horses (1).

A total of 16 studies reported the age of equine participants. A total of 3 studies categorized horses as foals and a further 9 reported horses as adults. Specific ages were reported in 10 of the 16 studies: 16 to 18 months (1); 5 to 13 years old (1); 2 to 24 years old (1); 6 to 13 years old (1); 8 to 20 years old (1); 22 years old; and 4 to 28 years old, with 2 studies reporting averages (i.e., means) of 7.4 years old (SD = 3.4); 14 years old (SD = 6.98); and 17.3 years old (SD = 5.7). A total of 16 studies reported on the sex of equine participants, described as follows: geldings and mares (7); geldings (2); geldings, mares, and stallions (2); colts and fillies (2); broodmares and stallions (1); females and geldings (1); and females and males (1). 

#### 3.2.2. Nature of HHIs

Horses interacted with humans in a variety of ways in the included studies. Handling was observed in 21 studies (46.6%), followed by riding in 11 studies (24.4%). A total of 4 studies described an interaction that did not involve physical contact between horses and humans (e.g., observation of behaviour in proximity to a human). Another 4 studies examined a combination of riding and handling interactions. A total of 3 studies investigated handling and grooming, 1 focused on riding, and 1 on training, exclusively. 

#### 3.2.3. Publication Years of Papers 

All 45 papers in the current review were published between the years 2002 and 2021. The greatest number of papers were published in 2018 (n = 8), followed by 5 each in 2017 and 2020. Only 1 paper was published before the year 2008. 

Included papers were published across a range of journals in veterinary health and medicine. Over a third (35.5%, n = 16) of papers were published in *Applied Animal Behaviour Science* and 5 papers were published by *Animals*, followed by three papers in *Physiology & Behavior*. A total of 2 papers each were published by the following seven journals: *Animal Cognition; Animal Science Journal; Behavioural Processes; Bulletin of University of Agricultural Sciences and Veterinary Medicine; Frontiers in Veterinary Science*; *Journal of Applied Animal Welfare Science; Society & Animals;* and *The Veterinary Journal*. Finally, 1 paper was published in each of the following seven journals: *Applied Animal Science; Anthrozoos; Bulletin of the Veterinary Institute; Early Child Development and Care; Journal of Equine Veterinary Science; Journal of Veterinary Behavior;* and *Journal of Veterinary Research.*

#### 3.2.4. Description of Studies

Both physiological and behavioural measures of horse response to human interactions were reported in 42.2% (n = 19) of studies; a further 31.1% (n = 14) exclusively used physiological measures and 26.6% (n = 12) used qualitative measures (i.e., behavioural observation). Only six papers described studies that included a control group or condition [38,39,40,41,42,43].

#### 3.2.5. Country of Study 

All 45 papers were published in English and available in full-text, though were conducted across 11 different countries. A total of 8 studies were conducted in France, followed by 6 each in Italy and Poland, 5 in Canada, and 4 in the United Kingdom. A total of 3 studies were conducted in the Netherlands and Sweden, and 2 in Japan, Romania, and the United States. Finally, 1 study was conducted in each of the following four countries: Australia, Germany, New Zealand, and Thailand.

### 3.3. Measurement of HHIs in the Horse

#### 3.3.1. Physiological Measures

Approximately three quarters of the studies in the present review used physiological measurements to explore the effect of the HHIs. Heart rate (HR) data was obtained in 27 studies [37,38,39,40,42,43,44,45,46,47,48,49,50,51,52,53,54,55,56,57,58]. Of these studies, 23 used the Polar HR monitor on horses alone, while 2 studies used the Polar HR monitor on horses and humans [38,45]. A total of 2 studies used a portable electrocardiogram (ECG) for collecting HR data [51,59] from horses. Of studies that collected HR data, 11 reported HR alone [37,38,40,42,44,45,49,50,54,55,56,60] and 9 reported HR and heart rate variability (HRV) measures [39,43,47,48,52,53,57,58,59,61,62,63,64].

Cortisol data was obtained in 9 studies [41,43,51,61,63,64,65,66,67]. A total of 4 studies collected blood samples [41,51,61,65] and 5 collected saliva samples [43,63,64,66,67] for measurements of cortisol concentrations. Finally, 3 studies collected other measurements including eye temperature [46,57], core temperature [45], plasma lactate concentrations [65,68], plasma β endorphin [65], adrenocorticotropic hormone (ACTH) concentrations [61,65], and muscle tone [66]. 

#### 3.3.2. Behavioural Measures

Two thirds of the studies in this review used behavioural observation measures to explore the effect of human interactions on the horse. Observations of equine behaviour consisted of direct observation in 19 studies (42.2%) [42,44,47,49,52,53,56,60,61,69,70,71,72,73,74,75,76,77,78], and remaining studies (n = 12) used video analysis [38,39,40,48,50,62,63,64,68,79,80,81].

Likert scales were used to describe equine behaviour in eight studies [40,47,48,49,64,68,76,79]. A total of 2 studies adapted scales described in the literature [47,68]. Only 1 study [76] developed a qualitative behaviour rating scale that originally consisted of 36 qualitative expressions and were narrowed down to 13 descriptions of horse behaviour by focus groups with horse professionals. Similarly, Minero et al. [76] used qualitative behavioural assessment with veterinarian observers to investigate the response of foals to unfamiliar humans. Development of descriptors for likert ratings was also described in Birke and Hockenhull [45]’s study on pairings with familiar and unfamiliar humans. External observers were asked to view video recordings of human–horse dyads and describe interactions in their own words. Transcripts were used to develop a word map, from which researchers used the four most frequent words (tension, cooperativeness, trust, and attention) to generate likert scales for a second panel of observers. Only 1 study used a self-reported survey, completed by horse owners, to understand HHIs [73].

Ethograms were explicitly described in 9 studies [38,39,42,48,50,52,53,58,61,72,75]. Blokhuis et al. [38] used an ethogram of observational behaviours related to horse discomfort, such as head-toss and rear, in connection to the position of the rider’s seat. Similarly, Mendonca [52] developed an ethogram to measure horses’ emotional state, consisting of the physical movements of horses (i.e., ears pinned, lateral head movement), vocal expressions (i.e., snorts), and defecation. Finally, Thorbergson et al. [42] developed a list of 32 horse behaviours that were separated into three groups (agitated, relaxed, and ambiguous) based on previous research. Coding of equine behaviour was described in other studies, often created for the purpose of the study [44,63]. Standardized behavioural tests were used in many of the studies in the current review. In many cases, these tests (e.g., motionless person test) were adapted to each individual study (e.g., [59,62,69,70,71,74]). For more information on the standardized tests used in studies, see 4.4.1 below.

### 3.4. Findings from Thematic Analysis

The thematic analysis is presented in an Excel spreadsheet in Table S3. All 39 papers were classified into six major themes:

1. Standardized Behavioural Tests
2. Incongruent Behavioural and Physiological Responses
3. Horse Emotional State and Response
4. Background and Experiences of Human Participants
5. Human–Horse Relationship and the “Buffering” Effect
6. Equine Welfare

#### 3.4.1. Theme 1: Standardized Behavioural Tests 

Repetition of HHI tests were observed across many studies in the current review. The voluntary animal approach test was used in three studies [69,70,71]. In this test, the latency time in seconds for a horse to approach a human who is standing still outside of its box is recorded. Similarly, the motionless person test assesses whether a horse approaches either a familiar or unfamiliar human who is standing still at a distance from the horse. The motionless person test was used in six studies [49,50,54,62,74,80]; two of these studies tested the effect of both familiar and unfamiliar humans [49,50]. 

The forced animal approach test was used in eight studies [49,50,69,70,71,74,76,77]; this test examined horse response to a human that approaches the horses. Similarly, the avoidance tests, which assesses the proximity that a human can reach to an equine before the animal moves away, was used in four studies [59,69,70,76]. Finally, the novel object tests, which assesses equine response to an unfamiliar or new object, was used in two studies [49,56].

#### 3.4.2. Theme 2: Incongruent Behavioural and Physiological Responses

Inconsistency between measures of equine behaviour and physiological response was noted in three studies [47,48,57]. Janczarek et al. [47] exposed horses to human physical contact over a six day period, where contact consisted of stroking different body regions (head, neck, trunk, front limbs, and hind limbs). Strokes were associated with greater excitability, as identified by increases in HR and HRV (r = 0.53, *p* < 0.05 during head strokes); however, behavioural changes (observations based on a scale of horse attitude), were not noted in relation to this physical contact. Stroking different regions of the horses’ bodies led to different physiological responses, depending on individual preferences. 

In contrast to these findings, Konig von Borstel et al. [48], observed that human interaction with horses (i.e., riding and leading) had a stronger effect on behavioural change, specifically reactivity and emotionality, than on HR and HRV. Finally, when horses were ridden through novel obstacles, Squibb et al. [57] noticed that physiological indicators of stress (i.e., heart rate (HR), heart rate variability (HRV), and eye temperature) were not associated with compliance. The researchers suggest that horses’ observable behaviour did not appear to reflect their psychological and physiological response to stress. 

#### 3.4.3. Theme 3: Horse Emotional State and Response 

The relationship between horses and humans and its effect on horse emotional regulation was a focus of multiple studies in this review [39,55,58,69,70]. Studies varied widely in their approach to measuring the emotional state of horses. While HR [49,50,54] and HRV [47,48,59] were used to measure emotional reactivity, most studies used both physiological and behavioural measures [47,48,49,50]. Only three studies [72,73,78] examined behavioural measures alone; specifically, whether frequency of snorts [78], horse muscle tension and posture [73], and other observable behaviours [72] correlated to the horses’ emotional state. 

Although HR and HRV were often used to determine horse emotional reactivity [54], the validity and reliability of physiological measures for reactivity was contested by one study [50]. Lansade and Bouissou [50] observed that HR did not correlate with previously supported behavioural indicators of reactivity and was not reliable over time; the researchers argue that HR is too sensitive and non-specific due to external influences beyond the experimenter’s control (e.g., noises or visual stimuli). 

The connection between physical touch of the horse and emotional reactivity was examined in three studies [47,49,59]. Janczarek et al. [47] observed that stroking was associated with greater excitability in horses, as identified by an increase in HR and HRV. Stroking different bodily regions led to different physiological responses, which researchers believe correspond to individual horses’ preferences; this finding was also observed by Kozak et al. [49]. Similarly, grooming led to lower HRV in Scopa et al. [59]. 

A sub-theme related to components of horse temperament was observed in three studies [48,49,50]. Kozak et al. [49] noted that emotional reactivity appears to be a trait consisting of multiple variables rather than one indicator of horse temperament. Fear reactivity to interaction with humans was found to be a key and stable component of horse temperament in one study [48], and a potentially stable “reactivity-to-humans” trait was observed in another [50].

Equine stress as a measure of reactivity to humans represented another sub-theme related to horse emotional state and regulation. Similar to studies examining emotional reactivity, most studies attributed change in physiological measures to observations of stress in the equine. These measures included HR and/or HRV [40,42,44,57,61,62], cortisol levels [41,65], and core eye temperature [57]. Familiarity with humans was shown in some studies to influence the stress response in horses [44]; specifically, horses demonstrated lower stress responses to familiar than to unfamiliar humans [59]. However, this was not substantiated in all studies [75]. 

#### 3.4.4. Theme 4: Background and Experiences of Human Participants

The majority of studies described adult human participants; however, eight papers described studies with more specific human populations. Children and youth were used in five studies [44,61,65,66,67], four of which included children with complex health care needs [61,65,66,67], and one with at-risk adolescents [44]. One study [51] described veterans diagnosed with post traumatic stress disorder, and another [40] examined the influence of patients with psychological and physical challenges. 

The humans used in studies had various levels of experience with horses. Twelve studies used experienced handlers [46,58,59,60,72,79,81], riders [38,45,68,78] and/or trainers [55]. Five studies used novices, specifically children and adolescents [44,61,65,66,67]. Three studies examined the influence of humans with a variety of experience with horses [41,53,54]. Approximately half of the included studies (n = 25, 55.5%) did not provide a description of the experience level of human participants.

#### 3.4.5. Theme 5: Human–Horse Relationship and the “Buffering” Effect 

Many studies in the current review referred to the potential of a “buffering” effect where the presence of a human was observed to result in diminished horse reactivity [58] and facilitated habituation [82]. In studies where detailed observations and descriptions of the relationship between horses and humans was provided, horses paired with familiar humans were observed to have a strong human–horse relationships evidenced by working together in a coordinated manner [79]. Conversely, unfamiliar humans led to detrimental observations of behavioural measures [79] of the human–horse bond. 

Studies using objective behavioral and physiological measures to evaluate the effect of humans on horses during HHIs also suggest a “buffering” effect [46,58] when humans were present, but this was not dependent on the human being familiar to the horse. Similarly, Hartman et al. [60] did not observe a change in equine behaviour, specifically ease of handling, as a function of handler familiarity. 

The perception of humans in general [70] and exposure over time [56,62] also appears to play a key role in the development of the human–horse relationship. In a study examining interactions between at-risk adolescents and horses in a therapy setting, Arrazol and Merkies [44] noted that human emotional and mental difficulties appeared to influence the horses’ perception of humans; however, over time, horses demonstrated improved social bonds to humans, suggesting that familiarity and exposure plays a key role in developing a strong human–horse bond [63,70]. Similarly, Visser et al. [58] noted an increase in heart rate and decrease in heart rate variability, which was more pronounced in untrained horses, suggesting a buffering of emotional reactivity when horses had previous experience with a handler/human.

Training experience may also have an important influence on the human–horse relationship, as observed in two studies [55,71]. Negative reinforcement [55] and traditional handling exercises, as opposed to natural horsemanship [71], were specifically found to negatively impact the human–horse relationship. These experiences, which resulted in a poor bond with humans, has led to concerns regarding safety and handling [76]. The generalizability of these findings, however, is not clear. In a study investigating the impact of stressful physical contact (i.e., grooming and handling) on the human–horse bond, Gorecka-Bruzda et al. [62] did not observe an impact on the human–horse bond if these experiences took place during the pre-weaning stage. 

Some studies have shown the possibility that the presence of a human can moderate the horse’s emotional response to various stimuli. Munsters et al. [68] observed a decrease in the heart rate of horses used for police riot work, to which they attributed to mean that the rider was able to mitigate the horses’ fear response. The importance of a good horse-rider match in reducing stress in ridden horses has also been demonstrated [53]. Furthermore, a behavioural observation of horses that were ridden and led showed that leading resulted in lower behavioural reactions, which was interpreted to mean that a handler on the ground may have a stronger influence on horses’ behaviour than when mounted [46]. 

#### 3.4.6. Theme 6: Equine Welfare 

The welfare of horses was the focal point of many studies in the current review. The effect of therapeutic sessions on equine participants was examined in four studies where welfare was assessed through stress levels [51,61,65,66]. Three of these studies [51,61,66] did not observe changes in cortisol concentration or HR in horses used for therapy sessions, suggesting that therapy may not be a stressful event for horses. One study [65] noted a higher stress response, as indicated by cortisol levels, in horses ridden by children with psycho-motor disabilities than healthy children. One explanation for these findings is that the training horses receive to become therapy horses may impact horse perception and emotional regulation, in effect influencing behavioural and physiological responses to stimuli [52]. Modifying horse perception to novel stimuli and regulating the behavioural and physiological response may require repeated exposure to a new environment, such as the therapeutic setting [44]. However, exposing horses to environments beyond the scope of their specific training, which may induce fear or require aggressive training techniques during exposure, should be avoided as this may have negative consequences for their overall welfare [52,68].

Handling techniques used by humans in other types of human horse interactions were identified as an important component of equine welfare. Costa et al. [69] noted through direct observations of horse behaviour that horses cared for in a “sub-optimal” environment demonstrated adverse behaviours (e.g., avoidance and aggression) towards all humans. Poor treatment by humans was also associated with unwanted behaviours in other studies [77]; for example, poor handling is associated with horse behaviours, including fear of humans as a function of greater arousal and aggressive behaviours (rτ = 0.6, *p* < 0.05). [76]. Moreover, positive versus negative reinforcement is associated with horse emotional reactivity [55]. Specifically, Sankey et al. [55] noted that positive reinforcement was observed to lead to increased, long-term interest in humans, whereas negative reinforcement led to increases in emotional reactivity as indicated by increases in HR and avoidance of human contact. 

## 4. Discussion

This scoping review aimed to explore how the effects of HHIs are measured in the horse and the known effects of these interactions on equine physiology and welfare. A total of 45 articles from eleven different countries were identified by the search strategy. Nearly all of the articles, with the exception of one, were published after the year 2008 when Hausberger et al. [5] published their seminal review on the human–horse relationship. Studies included a total of 1934 equine participants of diverse breeds, backgrounds, and ages. Interactions between humans and horses primarily consisted of handling (46.6%) and riding (24.4%). Remaining HHIs included a combination of riding and handling (8.9%), no physical contact (8.9%), handling and grooming (6.7), riding (2.2%), and training (2.2%). Measures of these interactions included behavioural observation and physiological measures, including HR, HRV, cortisol (blood and saliva), muscle tension, eye temperature, core temperature, plasma lactate concentrations, plasma β endorphin, and adrenocorticotropic hormone concentrations. Nearly half (42.2%) of the included studies used both behavioural observation and physiological measures in the assessment of HHIs. A further 26.6% only used behavioural observation and 31.1% exclusively used physiological measures. 

This review sought to identify the various ways that interactions between horses and humans are measured. Various practices of assessment and measurement of HHIs have been identified in the literature. In a previous review investigating the nature of HHIs, Hausberger et al. [5] noted that measurements of interactions fell into three categories: (1) observation (i.e., ratings of equine behaviour and/or personality); (2) behavioural tests and measures (i.e., standardized assessments and/or scores of reactivity); and (3) physiological measures (e.g., HR, HRV, and salivary and blood cortisol samples) [5]. This diversity in measurement of HHIs was also observed in the current study, whereby studies exclusively used behavioural or physiological measures, or a combination of observed or standardized behavioural assessment with physiological measures. Importantly, the majority of articles identified in this review (69%) were published since the previous review by Hausberger et al. [5] thus providing an update on the literature in this field.

More general reviews on human–animal interactions reveal the use of questionnaires, consisting of self-reports or subjective reporting by others [31,83,84]. The use of subjective reports was only observed in one study in the current review [73]. The ultimate goal of such assessments in determining the affective state of the animal and indicating whether the interaction is indeed positive, can be difficult to ascertain as it is based on the human perspective. The use of objective physiological measures provide an unbiased perspective that apparently represent the state of the horse. HHIs observed in the current review included led, ridden, and unrestrained interactions. Therefore, some interactions were imposed upon the horse, during which time behaviors and physiological measures were obtained to assess the horse’s “affective state”. With unrestrained, voluntary interactions, the horse had a choice to interact or not; however, behavioral observations were recorded without physiological measures for some of these interactions. In the reviewed studies, equine focused measurements included both behavioral and physiological measures yet only a few papers measured both during all types of interactions (see Table S4 for more information); this finding is supported by previous investigations [22]. 

### 4.1. Limitations 

This scoping review sought to provide an overview of current practices related to the measurement of HHIs and explore effects of these interactions on horse behaviour, physiology, and welfare. Due to limited research on the topic, the present synthesis covered a wide range of equine and human populations, allowing for learnings across different contexts. This may also be a limitation of the present study. The heterogeneity in equine participant breed, age, and use, may have contributed to the diversity in findings, which likely affect the generalizability of findings from this review. Moreover, unlike systematic reviews, scoping reviews do not assess the methodological rigor or quality of primary studies. Instead, they rely on the critical appraisal and interpretation of results in each of the assessed studies.

Despite our rigorous approach to article identification and evaluation, it is possible that some relevant articles may have been missed. More specifically, although every effort was made to capture articles that describe HHIs, it is unlikely that every relevant article was identified by the database search strategies. For example, the addition of the search terms “gelding” and “filly” may have led to the identification of additional papers. Moreover, interactions within the sport literature may have been inadvertedly missed due to a lack of specified keywords (e.g., polo). Finally, given the extensive use of horses across various settings, it is likely that some articles in the non-academic databases may not have been documented in this review. 

### 4.2. Gaps and Recommendations for Future Research 

This scoping review supports previous findings related to HHIs that current evidence and measurement practices in the literature are varied and heterogeneous [22]. To date, there appears to be little consensus regarding reliable and valid measures of horse emotional state and reaction to human interaction. The science of human–animal interaction is often criticized for lack of methodological rigor and use of standardized tools [5,31,83] and its subsequent influence on animal welfare [83]. Significant heterogeneity was observed between studies examining the effect of HHIs on horses, reflecting similar reviews on the topic [22]. This finding indicates a need for standardization in measurement and reporting to improve understanding on the impact of HHIs on the horse. To determine the effect of various human interactions on equine behaviour, physiology, and welfare, further research employing standardized assessment and objective inquiry are required. Based on this review, several gaps in the literature have been identified that need to be addressed. 

Many of the studies in the current review attempted to measure stress and concluded that lack of stress, based on physiological and behavioural indicators, was an indication of good welfare during human horse interactions. Although this is one component of welfare, positive experiences perceived by the animal are also an important aspect of animal welfare [85]. Therefore, more robust evaluations of welfare, including measurements of the horse’s affective state during human horse interactions, are warranted. This was also the recommendation in reviews by Hall et al. [6] and Merkies [17]. A more comprehensive evaluation will likely require the combined use of current methods along with addition of new methods; for example, through the continued use of physiological and behavioural measures of stress along with measures that assess a broader aspect of horse affective states. These could include ethograms with affiliative behaviors [86] and physiological measures of hormones of well-being such as oxytocin and serotonin [87]. Studies using a cognitive bias approach also show promise toward understanding animal emotion [88]. An emphasis on methods that use both behavioural and physiological measures is necessary since behavioural responses to the environment can be suppressed. Horses with passive coping styles [89] and horses who are well trained [17] may not readily show behaviours indicative of stress or aversion, while physiological measures continue to indicate sympathetic nervous system (SNS) stimulation. Further understanding of current methods is also important. Cortisol concentrations can reflect arousal and excitement as well physical activity. HRV measures, which reflect the parasympathetic and sympathetic aspects of the autonomic nervous system, are complex and require further knowledge including an understanding of nonlinear measures. Continued analysis of the relationship between behaviours and physiological measures of the equine affective state may lead to clear biomarkers for measurements of stress and well-being [90]. An improved ability to assess the horse’s emotional state during HHIs will require an expansion in the use and understanding of current research methods and discovery and implementation of new methods. Although this may be a difficult task, it will be critical in truly assessing horse welfare during horse human interactions and proposing future improvements towards equine welfare in the equine industry. 

## 5. Conclusions 

Ensuring the welfare of horses during HHIs is vital to promoting positive and safe relationships between humans and horses across various settings. This scoping review illustrates the diverse nature of HHIs and their measurement within the literature. Current evidence of equine welfare during HHIs is minimal and requires further investigation. For example, the assessment of equine welfare goes beyond the physical state of a horse and includes the emotional state of the animal; standardized approaches to measuring these aspects of welfare within the horse is needed to advance understanding of how interactions with humans impacts equine welfare. Moreover, current literature evaluating the emotional state of horses largely focuses on the absence of a negative affective state. Broadening the existing scope of methods to evaluate a positive affective state would improve the overall understanding of the horse’s welfare during HHIs. 

Research is essential to continue to advance our understanding of negative and positive affective states of horses, including the measurement and recognition of such emotional states; such research can continue to be used to inform policy makers in the equine industry. The practical application of knowledge gained through research needs to be addressed. Changes are apparent in the perception of animals by humans in the 21st century. An emphasis on animals as companions and promotion of the human animal bond (HAB) is leading to positive changes in for animals in society. While stakeholders in the companion animal industry are emphasizing the importance of the HAB, stakeholders in the equine industry lag behind. Because horses do not live in the house with humans, they are not often considered a family member. However, promotion of horses as companions, rather than simply a mechanism for fun, may improve the attention to welfare [91]. Many equestrians genuinely want a positive relationship with their horse [79]. Therefore, informing horse owners, trainers, and coaches that every HHI has a considerable effect in the enhancement or declination of the HAB could influence their behaviour. Providing equestrians with tools to measure the emotional state of the horse during various interactions will also be essential for better attention to welfare. To this end, future aims in research should also include development and implementation of methods that can be used by equine stakeholders, and leaders in the field of equine health and welfare should be early adopters in promoting the HAB with equestrians and horses.

## Figures and Tables

**Table 1:** Inclusion and exclusion criteria.

Inclusion Criteria	Exclusion Criteria
Studies that focus on HHIs.	Studies that focus on other animals (e.g., dogs) or where the primary focus is not on interactions between horses and humans.
Qualitative or quantitative studies that investigate the effect of HHIs on the horse.	Reviews, editorials.
Studies that examine the effect of HHIs in the horse (e.g., physiological responses, observations, etc.).	Studies that exclusively examine the effect of HHIs in the human (i.e., hippotherapy, equine-assisted/facilitated learning).
Studies published in English.	Studies published in any language other than English.
Full text available.	Conference proceedings or articles where full text is not available.
No limit on year of publication.	Studies of working horses in developing countries.

**Table 2:** Data extraction form.

Category	Extracted Component
Article information	Full article citation
	Country of study
	Article source (i.e., Medline, CAB Abstracts, PsychInfo database, other evidence database, hand search)
Methodological process	Aim of the study
	Study population and sample size, including description of equine participants (if applicable)
	Outcome measured
	Evaluation process (i.e., how the study was carried out, if applicable)
Description of HHIs	Type of human interaction (e.g., handling, riding, non-physical interaction, etc.)
	Measurement focus (e.g., observational, behavioral, physiological, etc.)
	Measurement tools (if applicable)
Key findings	Main study outcome as related to the effect of the bond in the horse (e.g., welfare), measurement of the bond, and/or description of the physiology of the bond
	Other key findings (if applicable)

## Supplementary Materials

The following are available online at https://www.mdpi.com/article/10.3390/ani11102782/s1, Table S1: Syntax for keyword search, Table S2: Grey literature search results, Table S3: Thematic analysis categorization, Table S4: Data extraction full results.

## References

[1] van Weeran R. (2017). Horses and humans: A special bond throughout the ages. ARGOS.

[2] Hausberger M., Muller C. (2002). A brief note on some possible factors involved in the reactions of horses to humans. Appl. Anim. Behav. Sci..

[3] Hawson L., McLean A., McGreevy P. (2010). The roles of equine ethology and applied learning theory in horse-related human injuries. J. Vet. Behav..

[4] Thompson K., McGreevy P., McManus P. (2015). A Critical Review of Horse-Related Risk: A Research Agenda for Safer Mounts, Riders and Equestrian Cultures. Animals.

[5] Hausberger M., Roche H., Henry S., Visser E.K. (2008). A review of the human-horse relationship. Appl. Anim. Behav. Sci..

[6] Hall C., Randle H., Pearson G., Preshaw L., Waran N. (2018). Assessing Equine Emotional State. Appl. Anim. Behav. Sci..

[7] Estep D., Hetts S., Davis H., Balfour D. (1992). Interactions, relationships, and bonds: The conceptual basis forscientist-animal relations. The Inevitable Bond: Examining Scientist-Animal Interactions.

[8] Butler D., Valenchon M., Annan R., Whay H.R., Mullan S. (2019). Living the ‘Best Life’ or ‘One Size Fits All’-Stakeholder perceptions of racehorse welfare. Animals.

[9] McDaniel P.B.C., Wood W. (2017). Autism and Equine-Assisted Interventions: A Systematic Mapping Review. J. Autism Dev. Disord..

[10] Boss L., Branson S., Hagan H., Krause-Parello C. (2019). A Systematic Review of Equine-Assisted Interventions in Military Veterans Diagnosed with PTSD. J. Veteran Stud..

[11] Kendall E., Maujean A., Pepping C.A., Downes M., Lakhani A., Byrne J., Macfarlane K. (2014). A systematic review of the efficacy of equine-assisted interventions on psychological outcomes. Eur. J. Psychother. Couns..

[12] Frewin K., Gardiner B. (2005). New age of old sage? A review of equine assisted psychotherapy. Aust. J. Couns. Psychol..

[13] Hoagwood K.E., Acri M., Morrissey M., Peth-Pierce R. (2018). Animal-assisted therapies for youth with or at risk for mental health problems: A systematic review. Appl. Dev. Sci..

[14] O’Haire M., Guerin N., Kirkham A. (2015). Animal-assisted intervention for trauma: A systematic literature review. Front. Psychol..

[15] van Houtert E., Endenburg N., Wijnker J., Rodenburg B., Vermetten E. (2018). The study of service dogs for veterans with post-traumatic stress disorder: A scoping literature review. Eur. J. Psychotraumatol..

[16] Mellor D. (2017). Operational Details of the Five Domains Model and Its Key Applications to the Assessment and Management of Animal Welfare. Animals.

[17] Merkies K., Franzin O. (2021). Enhanced Understanding of Horse–Human Interactions to Optimize Welfare. Animals.

[18] Scopa C., Contalbrigo L., Greco A., Lanatà A., Scilingo E.P., Baragli P. (2019). Emotional Transfer in Human–Horse Interaction: New Perspectives on Equine Assisted Interventions. Animals.

[19] Clayton M. (2012). What is entrainment? Definition and applications in musical research. Empir. Musicol. Rev..

[20] Payne E., DeAraugo J., Bennett P., McGreevy P. (2016). Exploring the existence and potential underpinnings of dog–human and horse–human attachment bonds. Behav. Process..

[21] Payne E., Boot M., Starling M., Henshalla C., McLeanb A., Bennettc P., McGreevy P. (2015). Evidence of horsemanship and dogmanship and their application in veterinary contexts. Vet. J..

[22] Clough H., Burford J., Roshier A., England G., Freeman S.L. (2019). A scoping review of the current literature exploring the nature of the horse-human relationship. Vet. Evid..

[23] Olczak K., Nowicki J., Klocek C. (2016). Motivation, stress and learning-critical characteristics that influence the horses’ value and training method-A review. Ann. Anim. Sci..

[24] Colquhoun H., Levac D., O’Brien K., Strausd S., Triccod A.C., Perrierd L., Kastnerd M., Moher D. (2014). Scoping reviews: Time for clarity in definition, methods, and reporting. J. Clin. Epidemiol..

[25] Tricco A., Lillie E., O’Brien K., O’Brien K.K. (2018). PRISMA extension for scoping reviews (PRISMA-ScR): Checklist and explanation. Ann. Intern. Med..

[26] Peters M.D.J., Godfrey C.M., McInterney P., Soares C., Khalil H., Parker D., Aromataris E., Munn Z. (2017). Scoping reviews. Institute Reviewers Manual.

[27] Khalil H., Peters M., Godfrey C., McInerney P., Soares C.B., Parker D. (2016). An evidence-based approach to scoping reviews. Worldviews Evid. -Based Nurs..

[28] Arksey H., O’Malley L. (2005). Scoping studies: Towards a methodological framework. Int. J. Soc. Res. Methodol..

[29] Joanna Briggs Institute (2014). Supporting Document for the Joanna Briggs Institute Levels of Evidence and Grades of Recommendation. https://joannabriggs.org/sites/default/files/2019–05/JBI%20Levels%20of%20Evidence%20Supporting%20Documents-v2.pdf.

[30] Ouzzani M., Hammady H., Fedorowicz Z., Elmagarmid A. (2016). Rayyan—A web and mobile app for systematic reviews. Syst. Rev..

[31] Rodriguez K., Guérin N., Gabriels R., Serpell J.A., Schreiner P.J., O’Haire M.E. (2018). The State of Assessment in Human-Animal Interaction Research. Hum. Anim. Interact. Bull..

[32] Hawson L. (2012). Compliance, cooperation, conditioning and cognition: Four Cs in the assessment of the horse-rider dyad. Vet. J..

[33] Roser M. (2014). Human Development Index (HDI). https://ourworldindata.org/human-development-index.

[34] McHugh M. (2012). Interrater reliability: The kappa statistic. Biochem. Med..

[35] Braun V., Clarke V. (2006). Using thematic analysis in psychology. Qual. Res. Psychol..

[36] Moher D., Liberati A., Tetzlaff J., Altman D.G. (2009). Preferred Reporting Items for Systematic Reviews and Meta-Analyses: The PRISMA Statement. PLoS Med..

[37] Pluta M., Osinski Z. (2014). Variability of heart rate in primitive horses and their relatives as an indicator of stress level, behavioural conduct towards humans and adaptation to living in wild. Bull. Vet. Inst. Pulawy.

[38] Blokhuis M.Z., Aronsson A., Hartmann E., Van Reenen C.G., Keeling L. (2008). Assessing the rider’s seat and horse’s behaviour: Difficulties and perspectives. J. Appl. Anim. Welf. Sci..

[39] Mendonca T., Bienboire-Frosini C., Menuge F., Leclercq J., Lafont-Lecuelle C., Arroub S., Pageat P. (2019). The Impact of Equine-Assisted Therapy on Equine Behavioral and Physiological Responses. Animals.

[40] Merkies K., Sievers A., Zakrajsek E., MacGregor H., Bergeron R., König von Borstel U. (2014). Preliminary results suggest an influence of psychological and physiological stress in humans on horse heart rate and behavior. J. Vet. Behav..

[41] Ono A., Matsuura A., Yamazaki Y., Sakai W., Watanabe K., Nakanowatari T., Kobayashi H., Irimajiri M., Hodate K. (2017). Influence of riders’ skill on plasma cortisol levels of horses walking on forest and field trekking courses. Anim. Sci. J..

[42] Thorbergson Z.W., Nielsen S.G., Beaulieu R.J., Doyle R.E. (2016). Physiological and behavioral responses of horses to wither scratching and patting the neck when under saddle. J. Appl. Anim. Welf. Sci..

[43] von Lewinski M., Biau S., Erber R., Ille N., Aurich J., Faure J.-M., Möstl E., Aurich C. (2013). Cortisol release, heart rate and heart rate variability in the horse and its rider: Different responses to training and performance. Vet. J..

[44] Arrazola A., Merkies K. (2020). Effect of human attachment style on horse behaviour and physiology during equine-assisted activities-a pilot study. Animals..

[45] Hockenhull J., Young T.J., Redgate S.E., Birke L. (2015). Exploring synchronicity in the heart rates of familiar and unfamiliar pairs of horses and humans undertaking an in-hand task. Anthrozoos.

[46] Ijichi C., Griffin K., Squibb K., Favier R. (2018). Stranger danger? An investigation into the influence of human-horse bond on stress and behaviour. Appl. Anim. Behav. Sci..

[47] Janczarek I., Stachurska A., Wilk I., Krakowski L., Przetacznik M., Zastrzezynska M., Kuna-Broniowska I. (2018). Emotional excitability and behaviour of horses in response to stroking various regions of the body. Anim. Sci. J..

[48] Von Konig B.U., Euent S., Graf P., König S., Gauly M. (2011). Equine behaviour and heart rate in temperament tests with or without rider or handler. Physiol. Behav..

[49] Kozak A., Zieba G., Tietza M., Rozempolska-Rucińska I. (2018). Consistency of emotional reactivity assessment results obtained in different behavioural tests. Appl. Anim. Behav. Sci..

[50] Lansade L., Bouissou M.-F. (2008). Reactivity to humans: A temperament trait of horses which is stable across time and situations. Appl. Anim. Behav. Sci..

[51] Malinowski K., Yee C., Tevlin J.M., Birks E.K., Durando M.M., Pournajafi-Nazarloo H., Cavaiola A.A., McKeever K.H. (2018). The effects of equine assisted therapy on plasma cortisol and oxytocin concentrations and heart rate variability in horses and measures of symptoms of post-traumatic stress disorder in veterans. J. Equine Vet. Sci..

[52] Mendonça T., Bienboire-Frosini C., Kowalczyk I., Leclercq J., Arroub S., Pageat P. (2019). Equine activities influence horses’ responses to different stimuli: Could this have an impact on equine welfare?. Animals.

[53] Munsters C., Visser K., van den Broek J., Sloet van Oldruitenborgh-Oosterbaan M.M. (2012). The influence of challenging objects and horse-rider matching on heart rate, heart rate variability and behavioural score in riding horses. Vet. J..

[54] Pluta M., Kedzierski W. (2018). Emotional responses of horses to patients requiring therapy. Anim. Soc..

[55] Sankey C., Richard-Yris M.-A., Henry S., Fureix C., Nassur F., Hausberger M. (2010). Reinforcement as a mediator of the perception of humans by horses (*Equus caballus*). Anim. Cogn..

[56] Sondergaard E., Jago J. (2010). The effect of early handling of foals on their reaction to handling, humans and novelty, and the foal–mare relationship. Appl. Anim. Behav. Sci..

[57] Squibb K., Griffin K., Favier R., Ijichi C. (2018). Poker Face: Discrepancies in behaviour and affective states in horses during stressful handling procedures. Appl. Anim. Behav. Sci..

[58] Visser E.K., van Reenan C.G., van der Werf J.T.N., Schilder M.B.H., Knaap J.H., Barneveld A., Blokhuis H.J. (2002). Heart rate and heart rate variability during a novel object test and a handling test in young horses. Physiol. Behav..

[59] Scopa C., Greco A., Contalbrigo L., Fratini E., Lanata A., Scilingo E.P., Baragli P. (2020). Inside the interaction: Contact with familiar humans modulates heart rate variability in horses. Front. Vet. Sci..

[60] Hartmann E., Rehn T., Christensen J.W., Nielsen P.P., McGreevy P. (2021). From the horse’s perspective: Investigating attachment behaviour and the effect of training method on fear reactions and ease of handling-a pilot study. Animals.

[61] Contalbrigo L., Borgi M., De Santis M., Collascchi B., Tuozzi A., Toson M., Redaelli V., Odore R., Vercelli C., Stefani A. (2021). Equine-Assisted Interventions (EAIs) for Children with Autism Spectrum Disorders (ASD): Behavioural and Physiological Indices of Stress in Domestic Horses (*Equus caballus*) during Riding Sessions. Animals.

[62] Górecka-Bruzda A., Jaworski Z., Suwala M., Boron M. (2017). Longitudinal study on human-related behaviour in horses—Can horses (*Equus caballus*) be de-domesticated?. Appl. Anim. Behav. Sci..

[63] Górecka-Bruzda A., Jaworski Z., Suwala M., Sobczyńska M., Jastrzębska E., Ogłuszka M., Sankey C., Boroń M., Jezierski T. (2017). Aversiveness of husbandry procedures for pre-weaned foals: A comparison using behavioural and physiological indices. Appl. Anim. Behav. Sci..

[64] Merkies K., McKechnie M.J., Zakrajsek E. (2018). Behavioural and physiological responses of therapy horses to mentally traumatized humans. Appl. Anim. Behav. Sci..

[65] Fazio E., Medica P., Cravana C., Ferlazzo A. (2013). Hypothalamic-pituitary-adrenal axis responses of horses to therapeutic riding program: Effects of different riders. Physiol. Behav..

[66] Nuchprayoon N., Arya N., Ritruechai P. (2017). Stress cortisol and muscle stiffness in horses used for equine-assisted therapy. J. Appl. Anim. Sci..

[67] Yorke J., Nugent W., Strand E., Bolen R., New J., Davis C. (2013). Equine-assisted therapy and its impact on cortisol levels of children and horses: A pilot study and meta-analysis. Early Child Dev. Care.

[68] Munsters C., van den Broek J., van Weeren R., van Oldruitenborgh-Oosterbaan M.M.S. (2013). The effects of transport, riot control training and night patrols on the workload and stress of mounted police horses. Appl. Anim. Behav. Sci..

[69] Costa E.D., Dai F., Murray L.A.M., Guazzetti S., Canali E., Minero M. (2015). A study on validity and reliability of on-farm tests to measure human–animal relationship in horses and donkeys. Appl. Anim. Behav. Sci..

[70] Diugan E.-A., Spinu M., Popescu S. Human-animal relationship assessment in horses (*Equus caballus*) with different uses. Bull. UASVM Vet. Med..

[71] Fureix C., Pages M., Bon R., Lassalle J.-M., Kuntz P., Gonzalez G. (2009). A preliminary study of the effects of handling type on horses’ emotional reactivity and the human-horse relationship. Behav. Process..

[72] Goldhawk C., Grandin T., Pajor E. (2020). Effect of animal’s experience and rodeo procedures on behaviour of bucking horses at a large commercial rodeo in Canada. Appl. Anim. Behav. Sci..

[73] Kieson E., Felix C., Webb S., Abramson C.I. (2020). The effects of a choice test between food rewards and human interaction in a herd of domestic horses of varying breeds and experiences. Appl. Anim. Behav. Sci..

[74] Ligout S., Bouissou M.F., Boivin X. (2008). Comparison of the effects of two different handling methods on the subsequent behaviour of Anglo-Arabian foals toward humans and handling. Appl. Anim. Behav. Sci..

[75] Lundberg P., Hartmann E., Roth L.S.V. (2020). Does training style affect the human-horse relationship? Asking the horse in a separation-Reunion experiment with the owner and a stranger. Appl. Anim. Behav. Sci..

[76] Minero M., Dalla C.E., Dai F., Canali E., Barbieri S., Zanella A., Pascuzzo R., Wemelsfelder F. (2018). Using qualitative behaviour assessment (QBA) to explore the emotional state of horses and its association with human-animal relationship. Appl. Anim. Behav. Sci..

[77] Popescu S., Borda C., Oros D., Sandru D.C., Spinu M., Giupina R., Diugan E. (2016). Human-animal relationship: A comparative study in working and breeding horses. Bull. UASVM Vet. Med..

[78] Stomp M., Masson A., Henry S., Hausberger M., Lesimple C. (2020). Could snorts inform us on how horses perceive riding?. Behav. Process..

[79] Birke L., Hockenhull J. (2015). Journeys together: Horses and humans in partnership. Soc. Anim..

[80] Minero M., Vittoria T.M., Canli E., Wemelsfelder F. (2009). Quantitative and qualitative assessment of the response of foals to the presence of an unfamiliar human. Appl. Anim. Behav. Sci..

[81] Ringhofer M., Yamamoto S. (2017). Domestic horses send signals to humans when they face with an unsolvable task. Anim. Cogn..

[82] Górecka A., Bakuniak M., Chruszczewski M.H., Jezierski T. (2007). A note on the habituation to novelty in horses: Handler effect. Anim. Sci. Pap. Rep..

[83] Hosey G., Melfi V. (2014). Human-animal interactions, relationships and bonds: A review and analysis of literature. Int. J. Comp. Psychol..

[84] Herzog H.A. (2015). Gender Differences in Human–Animal Interactions: A Review. Anthrozoos.

[85] Mellor D. (2016). Updating Animal Welfare Thinking: Moving beyond the “Five Freedoms” towards “A Life Worth Living”. Animals.

[86] Pearson G., Waran N., Reardon R.J.M., Keen J., Dwyer C.M. (2021). A Delphi study to determine expert consensus on the behavioural indicators of stress in horses undergoing veterinary care. Appl. Anim. Behav. Sci..

[87] Lee G., Yoon M. (2021). Association of plasma concentrations of oxytocin, vasopressin, and serotonin with docility and friendliness of horses. Domest. Anim. Endocrinol..

[88] Mendl M., Burman O.H.P., Parker R., Paul E.S. (2009). Cognitive bias as an indicator of animal emotion and welfare: Emerging evidence and underlying mechanisms. Appl. Anim. Behav. Sci..

[89] Budzynski M. (2014). Stress reactivity and coping in horse adaptation to environment. J. Equine Vet. Sci..

[90] Contreras-Aguilar M.D., Henry S., Coste C., Tecles F., Escribano D., Ceron J.J., Hausberger M. (2019). Changes in Saliva Analytes Correlate with Horses’ Behavioural Reactions to An Acute Stressor: A Pilot Study. Animals.

[91] Bidda J., McGreevy P.D. (2010). Ethical equitation: Applying a cost-benefit approach. J. Vet. Behav..

