# Supplementary material for: The Effect of Human–Horse Interactions on Equine Behaviour, Physiology, and Welfare: A Scoping Review
Source: Animals (Basel). 2021 Sep 24;11(10):2782. doi: 10.3390/ani11102782 (PMC8532845; doi:10.3390/ani11102782)
Supplement: Supplementary file 1 [file animals-11-02782-s001.zip › Table S1 Syntax for KW searches.pdf]

Supplementary Materials 1

PsycINFO via EBSCOhost, September 1, 2020

Search #	Query	Limiters/Expanders	Last Run Via	Results
S23	S3 AND S18 AND S20	Limiters - Publication Year: 2019-2020 Expanders - Apply equivalent subjects Search modes - Find all my search terms	Interface - EBSCOhost Research Databases Search Screen - Advanced Search Database - PsycINFO	20
S22	S3 AND S18 AND S20	Limiters - Publication Year: 2019-2020 Expanders - Apply equivalent subjects Search modes - Find all my search terms	Interface - EBSCOhost Research Databases Search Screen - Advanced Search Database - PsycINFO	20
S21	S3 AND S18 AND S20	Expanders - Apply equivalent subjects Search modes - Find all my search terms	Interface - EBSCOhost Research Databases Search Screen - Advanced Search Database - PsycINFO	153
S20	S4 OR S5 OR S6 OR S7 OR S8 OR S9 OR S19	Expanders - Apply equivalent subjects Search modes - Find all my search terms	Interface - EBSCOhost Research Databases Search Screen - Advanced Search Database - PsycINFO	4,477
S19	DE "Interspecies	Expanders - Apply	Interface -	4,262

	Interaction"	equivalent subjects Search modes - Find all my search terms	EBSCOhost Research Databases Search Screen - Advanced Search Database - PsycINFO	
S18	S10 OR S11 OR S12 OR S13 OR S14 OR S15 OR S16 OR S17	Expanders - Apply equivalent subjects Search modes - Find all my search terms	Interface - EBSCOhost Research Databases Search Screen - Advanced Search Database - PsycINFO	321,318
S17	TI ("Stress" OR "Physiological Stress" OR "Psychological Stress" OR "Social Stress" OR "Stress Reactions" OR "Stress Reactions") OR AB ("Stress" OR "Physiological Stress" OR "Psychological Stress" OR "Social Stress" OR "Stress Reactions" OR "Stress Reactions")	Expanders - Apply equivalent subjects Search modes - Find all my search terms	Interface - EBSCOhost Research Databases Search Screen - Advanced Search Database - PsycINFO	207,298
S16	TI ("Animal Behavior" OR "Alarm Responses" OR "Animal Communication" OR "Animal Defensive Behavior" OR "Animal Emotions" OR "Animal Environments" OR "Animal Exploratory Behavior" OR "Animal Feeding Behavior" OR "Animal Grooming Behavior" OR "Animal Learning" OR "Animal Open Field Behavior" OR "Animal Personality" OR "Animal Physical	Expanders - Apply equivalent subjects Search modes - Find all my search terms	Interface - EBSCOhost Research Databases Search Screen - Advanced Search Database - PsycINFO	4,027

	Appearance" OR "Animal Play" OR "Animal Social Behavior") OR AB ("Animal Behavior" OR "Alarm Responses" OR "Animal Communication" OR "Animal Defensive Behavior" OR "Animal Emotions" OR "Animal Environments" OR "Animal Exploratory Behavior" OR "Animal Feeding Behavior" OR "Animal Grooming Behavior" OR "Animal Learning" OR "Animal Open Field Behavior" OR "Animal Personality" OR "Animal Physical Appearance" OR "Animal Play" OR "Animal Social Behavior")			
S15	TI ("Animal Welfare" OR "Animal Cruelty" OR "Animal Rights" OR "Animal Welfare Assessment") OR AB ("Animal Welfare" OR "Animal Cruelty" OR "Animal Rights" OR "Animal Welfare Assessment")	Expanders - Apply equivalent subjects Search modes - Find all my search terms	Interface - EBSCOhost Research Databases Search Screen - Advanced Search Database - PsycINFO	1,720
S14	TI ("Heart Rate" OR "Heart Rate Variability") OR AB ("Heart Rate" OR "Heart Rate Variability")	Expanders - Apply equivalent subjects Search modes - Find all my search terms	Interface - EBSCOhost Research Databases Search Screen - Advanced Search Database - PsycINFO	21,260
S13	DE "Heart Rate" OR DE "Heart Rate Variability"	Expanders - Apply equivalent subjects Search modes - Find	Interface - EBSCOhost Research	19,768

		all my search terms	Databases Search Screen - Advanced Search Database - PsycINFO	
S12	DE "Animal Behavior" OR DE "Alarm Responses" OR DE "Animal Communication" OR DE "Animal Defensive Behavior" OR DE "Animal Emotions" OR DE "Animal Environments" OR DE "Animal Exploratory Behavior" OR DE "Animal Feeding Behavior" OR DE "Animal Grooming Behavior" OR DE "Animal Learning" OR DE "Animal Open Field Behavior" OR DE "Animal Personality" OR DE "Animal Physical Appearance" OR DE "Animal Play" OR DE "Animal Social Behavior" OR DE "Attachment Behavior"	Expanders - Apply equivalent subjects Search modes - Find all my search terms	Interface - EBSCOhost Research Databases Search Screen - Advanced Search Database - PsycINFO	74,856
S11	DE "Stress" OR DE "Physiological Stress" OR DE "Psychological Stress" OR DE "Social Stress" OR DE "Stress Reactions" OR DE "Stress Reactions"	Expanders - Apply equivalent subjects Search modes - Find all my search terms	Interface - EBSCOhost Research Databases Search Screen - Advanced Search Database - PsycINFO	83,482
S10	DE "Animal Welfare" OR DE "Animal Cruelty" OR DE "Animal Rights" OR DE "Animal Welfare Assessment"	Expanders - Apply equivalent subjects Search modes - Find all my search terms	Interface - EBSCOhost Research Databases Search Screen - Advanced	3,458

			Search Database - PsycINFO	
S9	TI ((horse n1 human) n3 (relationship* or dyad* or interact* or bond* or communicat* or attachment)) OR AB ((horse n1 human) n3 (relationship* or dyad* or interact* or bond* or communicat* or attachment))	Expanders - Apply equivalent subjects Search modes - Find all my search terms	Interface - EBSCOhost Research Databases Search Screen - Advanced Search Database - PsycINFO	90
S8	TI ("horse rider" n1 (relationship* or dyad* or interact* or bond* or communicat*) OR ("horse rider" n1 (relationship* or dyad* or interact* or bond* or communicat*)	Expanders - Apply equivalent subjects Search modes - Find all my search terms	Interface - EBSCOhost Research Databases Search Screen - Advanced Search Database - PsycINFO	8
S7	TI (("equine assisted" n1 (therapy or intervention)) OR AB ("equine assisted" n1 (therapy or intervention))	Expanders - Apply equivalent subjects Search modes - Find all my search terms	Interface - EBSCOhost Research Databases Search Screen - Advanced Search Database - PsycINFO	74
S6	TI (equin* n2 (activit* or therapy)) OR AB (equin* n2 (activit* or therapy))	Expanders - Apply equivalent subjects Search modes - Find all my search terms	Interface - EBSCOhost Research Databases Search Screen - Advanced Search Database - PsycINFO	154
S5	TI ((horse* or equin*) N1 lead*) OR AB ((horse* or equin*) N1 lead*)	Expanders - Apply equivalent subjects Search modes - Find	Interface - EBSCOhost Research	29

		all my search terms	Databases Search Screen - Advanced Search Database - PsycINFO	
S4	TI ((horse* or equin*) N1 groom*) OR AB ((horse* or equin*) N1 groom*)	Expanders - Apply equivalent subjects Search modes - Find all my search terms	Interface - EBSCOhost Research Databases Search Screen - Advanced Search Database - PsycINFO	4
S3	S1 OR S2	Expanders - Apply equivalent subjects Search modes - Find all my search terms	Interface - EBSCOhost Research Databases Search Screen - Advanced Search Database - PsycINFO	5,531
S2	DE Horses	Expanders - Apply equivalent subjects Search modes - Find all my search terms	Interface - EBSCOhost Research Databases Search Screen - Advanced Search Database - PsycINFO	1,371
S1	TI (equin* or horse* or colt* or foal* or mare* or racehorse* or stallion*) OR AB (equin* or horse* or colt * or foal* or mare * or racehorse * or stallion *)	Expanders - Apply equivalent subjects Search modes - Find all my search terms	Interface - EBSCOhost Research Databases Search Screen - Advanced Search Database - PsycINFO	5,463

PsycINFO via EBSCOhost, August 13, 2019

Search #	Query	Limiters/Expanders	Last Run Via	Results
S21	S3 AND S18 AND S20	Expanders - Apply equivalent subjects Search modes - Find all my search terms	Interface - EBSCOhost Research Databases Search Screen - Advanced Search Database - PsycINFO	136
S20	S4 OR S5 OR S6 OR S7 OR S8 OR S9 OR S19	Expanders - Apply equivalent subjects Search modes - Find all my search terms	Interface - EBSCOhost Research Databases Search Screen - Advanced Search Database - PsycINFO	4,198
S19	DE "Interspecies Interaction"	Expanders - Apply equivalent subjects Search modes - Find all my search terms	Interface - EBSCOhost Research Databases Search Screen - Advanced Search Database - PsycINFO	4,009
S18	S10 OR S11 OR S12 OR S13 OR S14 OR S15 OR S16 OR S17	Expanders - Apply equivalent subjects Search modes - Find all my search terms	Interface - EBSCOhost Research Databases Search Screen - Advanced Search Database - PsycINFO	304,813
S17	TI ("Stress" OR "Physiological Stress" OR	Expanders - Apply equivalent subjects	Interface - EBSCOhost	195,689

	"Psychological Stress" OR "Social Stress" OR "Stress Reactions" OR "Stress Reactions") OR AB ("Stress" OR "Physiological Stress" OR "Psychological Stress" OR "Social Stress" OR "Stress Reactions" OR "Stress Reactions")	Search modes - Find all my search terms	Research Databases Search Screen - Advanced Search Database - PsycINFO	
S16	TI ("Animal Behavior" OR "Alarm Responses" OR "Animal Communication" OR "Animal Defensive Behavior" OR "Animal Emotions" OR "Animal Environments" OR "Animal Exploratory Behavior" OR "Animal Feeding Behavior" OR "Animal Grooming Behavior" OR "Animal Learning" OR "Animal Open Field Behavior" OR "Animal Personality" OR "Animal Physical Appearance" OR "Animal Play" OR "Animal Social Behavior") OR AB ("Animal Behavior" OR "Alarm Responses" OR "Animal Communication" OR "Animal Defensive Behavior" OR "Animal Emotions" OR "Animal Environments" OR "Animal Exploratory Behavior" OR "Animal Feeding Behavior" OR "Animal Grooming Behavior" OR "Animal Learning" OR "Animal Open Field Behavior" OR "Animal Personality" OR "Animal Physical Appearance" OR "Animal Play" OR "Animal Social Behavior")	Expanders - Apply equivalent subjects Search modes - Find all my search terms	Interface - EBSCOhost Research Databases Search Screen - Advanced Search Database - PsycINFO	3,866

S15	TI ("Animal Welfare" OR "Animal Cruelty" OR "Animal Rights" OR "Animal Welfare Assessment") OR AB ("Animal Welfare" OR "Animal Cruelty" OR "Animal Rights" OR "Animal Welfare Assessment")	Expanders - Apply equivalent subjects Search modes - Find all my search terms	Interface - EBSCOhost Research Databases Search Screen - Advanced Search Database - PsycINFO	1,606
S14	TI ("Heart Rate" OR "Heart Rate Variability") OR AB ("Heart Rate" OR "Heart Rate Variability")	Expanders - Apply equivalent subjects Search modes - Find all my search terms	Interface - EBSCOhost Research Databases Search Screen - Advanced Search Database - PsycINFO	20,378
S13	DE "Heart Rate" OR DE "Heart Rate Variability"	Expanders - Apply equivalent subjects Search modes - Find all my search terms	Interface - EBSCOhost Research Databases Search Screen - Advanced Search Database - PsycINFO	19,118
S12	DE "Animal Behavior" OR DE "Alarm Responses" OR DE "Animal Communication" OR DE "Animal Defensive Behavior" OR DE "Animal Emotions" OR DE "Animal Environments" OR DE "Animal Exploratory Behavior" OR DE "Animal Feeding Behavior" OR DE "Animal Grooming Behavior" OR DE "Animal Learning" OR DE "Animal Open Field Behavior" OR	Expanders - Apply equivalent subjects Search modes - Find all my search terms	Interface - EBSCOhost Research Databases Search Screen - Advanced Search Database - PsycINFO	74,105

	DE "Animal Personality" OR DE "Animal Physical Appearance" OR DE "Animal Play" OR DE "Animal Social Behavior" OR DE "Attachment Behavior"			
S11	DE "Stress" OR DE "Physiological Stress" OR DE "Psychological Stress" OR DE "Social Stress" OR DE "Stress Reactions" OR DE "Stress Reactions"	Expanders - Apply equivalent subjects Search modes - Find all my search terms	Interface - EBSCOhost Research Databases Search Screen - Advanced Search Database - PsycINFO	78,875
S10	DE "Animal Welfare" OR DE "Animal Cruelty" OR DE "Animal Rights" OR DE "Animal Welfare Assessment"	Expanders - Apply equivalent subjects Search modes - Find all my search terms	Interface - EBSCOhost Research Databases Search Screen - Advanced Search Database - PsycINFO	3,214
S9	TI ((horse n1 human) n3 (relationship* or dyad* or interact* or bond* or communicat* or attachment)) OR AB ((horse n1 human) n3 (relationship* or dyad* or interact* or bond* or communicat* or attachment))	Expanders - Apply equivalent subjects Search modes - Find all my search terms	Interface - EBSCOhost Research Databases Search Screen - Advanced Search Database - PsycINFO	83
S8	TI ("horse rider" n1 (relationship* or dyad* or interact* or bond* or communicat*) OR ("horse rider" n1 (relationship* or dyad* or interact* or bond* or communicat*)	Expanders - Apply equivalent subjects Search modes - Find all my search terms	Interface - EBSCOhost Research Databases Search Screen - Advanced Search Database -	7

			PsycINFO	
S7	TI (("equine assisted" n1 (therapy or intervention)) OR AB ("equine assisted" n1 (therapy or intervention))	Expanders - Apply equivalent subjects Search modes - Find all my search terms	Interface - EBSCOhost Research Databases Search Screen - Advanced Search Database - PsycINFO	64
S6	TI (equin* n2 (activit* or therapy)) OR AB (equin* n2 (activit* or therapy))	Expanders - Apply equivalent subjects Search modes - Find all my search terms	Interface - EBSCOhost Research Databases Search Screen - Advanced Search Database - PsycINFO	133
S5	TI ((horse* or equin*) N1 lead*) OR AB ((horse* or equin*) N1 lead*)	Expanders - Apply equivalent subjects Search modes - Find all my search terms	Interface - EBSCOhost Research Databases Search Screen - Advanced Search Database - PsycINFO	26
S4	TI ((horse* or equin*) N1 groom*) OR AB ((horse* or equin*) N1 groom*)	Expanders - Apply equivalent subjects Search modes - Find all my search terms	Interface - EBSCOhost Research Databases Search Screen - Advanced Search Database - PsycINFO	3
S3	S1 OR S2	Expanders - Apply equivalent subjects Search modes - Find all my search terms	Interface - EBSCOhost Research Databases Search Screen	5,319

			- Advanced Search Database - PsycINFO	
S2	DE Horses	Expanders - Apply equivalent subjects Search modes - Find all my search terms	Interface - EBSCOhost Research Databases Search Screen - Advanced Search Database - PsycINFO	1,291
S1	TI (equin* or horse* or colt* or foal* or mare* or racehorse* or stallion*) OR AB (equin* or horse* or colt * or foal* or mare * or racehorse * or stallion *)		Interface - EBSCOhost Research Databases Search Screen - Advanced Search Database - PsycINFO	5,253

CAB Abstracts via EBSCOhost, September 1, 2020

Search #	Query	Limiters/Expanders	Last Run Via	Results
S19	S3 AND S16 AND S17	Limiters - Publication Year: 2019-2020 Expanders - Apply equivalent subjects Search modes - Find all my search terms	Interface - EBSCOhost Research Databases Search Screen - Advanced Search Database - CAB Abstracts	33
S18	S3 AND S16 AND S17	Expanders - Apply equivalent subjects Search modes - Find all my search terms	Interface - EBSCOhost Research Databases Search	174

			Screen - Advanced Search Database - CAB Abstracts	
S17	S4 OR S5 OR S6 OR S7 OR S8 OR S15	Expanders - Apply equivalent subjects Search modes - Find all my search terms	Interface - EBSCOhost Research Databases Search Screen - Advanced Search Database - CAB Abstracts	934
S16	S9 OR S10 OR S11 OR S12 OR S13 OR S14	Expanders - Apply equivalent subjects Search modes - Find all my search terms	Interface - EBSCOhost Research Databases Search Screen - Advanced Search Database - CAB Abstracts	545,465
S15	TI ((horse n1 human) n3 (relationship* or dyad* or interact* or bond* or communicat*)) OR AB ((horse n1 human) n3 relationship* or dyad* or interact* or bond* or communicat*))	Expanders - Apply equivalent subjects Search modes - Find all my search terms	Interface - EBSCOhost Research Databases Search Screen - Advanced Search Database - CAB Abstracts	91
S14	TI ("Heart rate" OR "heart rate variability" or hydrocortisone) OR AB	Expanders - Apply equivalent subjects Search modes - Find	Interface - EBSCOhost Research	22,076

	(“Heart rate” OR “heart rate variability” or hydrocortisone)	all my search terms	Databases Search Screen - Advanced Search Database - CAB Abstracts	
S13	TI (Emotional n1 (health OR reaction)) OR AB (Emotional n1 (health OR reaction))	Expanders - Apply equivalent subjects Search modes - Find all my search terms	Interface - EBSCOhost Research Databases Search Screen - Advanced Search Database - CAB Abstracts	744
S12	TI (stress* OR fear*) OR AB (stress* OR fear*)	Expanders - Apply equivalent subjects Search modes - Find all my search terms	Interface - EBSCOhost Research Databases Search Screen - Advanced Search Database - CAB Abstracts	399,299
S11	TI (animal N1 (welfare OR well-being OR wellbeing OR wellness)) OR AB (animal N1 (welfare OR well being OR wellbeing OR wellness))	Expanders - Apply equivalent subjects Search modes - Find all my search terms	Interface - EBSCOhost Research Databases Search Screen - Advanced Search Database - CAB Abstracts	14,292
S10	TI ("animal welfare" OR	Expanders - Apply	Interface -	4,704

	"stress OR psychological needs" OR "animal behavio#r") OR AB ("animal welfare" OR "stress OR “psychological needs" OR "animal behavio#r")	equivalent subjects Search modes - Find all my search terms	EBSCOhost Research Databases Search Screen - Advanced Search Database - CAB Abstracts	
S9	SU "animal welfare" OR SU "stress" OR SU “stress factors” OR SU "psychological needs" OR SU "animal behaviour" OR SU “basic needs” OR SU “hydrocortisone” OR SU "heart rate"	Expanders - Apply equivalent subjects Search modes - Find all my search terms	Interface - EBSCOhost Research Databases Search Screen - Advanced Search Database - CAB Abstracts	331,995
S8	TI ("horse rider" n1 (relationship* or dyad* or interact* or bond* or communicat*) OR ("horse rider" n1 (relationship* or dyad* or interact* or bond* or communicat*)	Expanders - Apply equivalent subjects Search modes - Find all my search terms	Interface - EBSCOhost Research Databases Search Screen - Advanced Search Database - CAB Abstracts	20
S7	TI (("equine assisted" n1 (therapy or intervention)) OR AB ("equine assisted" n1 (therapy or intervention))	Expanders - Apply equivalent subjects Search modes - Find all my search terms	Interface - EBSCOhost Research Databases Search Screen - Advanced Search Database - CAB Abstracts	29

S6	TI (equin* n2 (activit* or therapy)) OR AB (equin* n2 (activit* or therapy))	Expanders - Apply equivalent subjects Search modes - Find all my search terms	Interface - EBSCOhost Research Databases Search Screen - Advanced Search Database - CAB Abstracts	642
S5	TI ((horse* or equin*) N1 lead*) OR AB ((horse* or equin*) N1 lead*)	Expanders - Apply equivalent subjects Search modes - Find all my search terms	Interface - EBSCOhost Research Databases Search Screen - Advanced Search Database - CAB Abstracts	166
S4	TI ((horse* or equin*) N1 groom*) OR AB ((horse* or equin*) N1 groom*)	Expanders - Apply equivalent subjects Search modes - Find all my search terms	Interface - EBSCOhost Research Databases Search Screen - Advanced Search Database - CAB Abstracts	16
S3	S1 OR S2	Expanders - Apply equivalent subjects Search modes - Find all my search terms	Interface - EBSCOhost Research Databases Search Screen - Advanced Search Database - CAB	158,163

			Abstracts	
S2	SU "horses" OR SU "colts" OR SU "foals" OR SU "mares" OR SU "racehorses" OR SU "stallions"	Expanders - Apply equivalent subjects Search modes - Find all my search terms	Interface - EBSCOhost Research Databases Search Screen - Advanced Search Database - CAB Abstracts	117,032
S1	TI (equin* or horse* or colt* or foal* or mare* or racehorse* or stallion*) OR AB (equin* or horse* or colt* or foal* or mare* or racehorse* or stallion*)	Expanders - Apply equivalent subjects Search modes - Find all my search terms	Interface - EBSCOhost Research Databases Search Screen - Advanced Search Database - CAB Abstracts	143,493

CAB Abstracts via EBSCOhost, August 13, 2019

Search #	Query	Limiters/Expanders	Last Run Via	Results
S18	S3 AND S16 AND S17	Expanders - Apply equivalent subjects Search modes - Find all my search terms	Interface - EBSCOhost Research Databases Search Screen - Advanced Search Database - CAB Abstracts	145
S17	S4 OR S5 OR S6 OR S7 OR S8 OR S15	Expanders - Apply equivalent subjects Search modes - Find all my search terms	Interface - EBSCOhost Research Databases	878

			Search Screen - Advanced Search Database - CAB Abstracts	
S16	S9 OR S10 OR S11 OR S12 OR S13 OR S14	Expanders - Apply equivalent subjects Search modes - Find all my search terms	Interface - EBSCOhost Research Databases Search Screen - Advanced Search Database - CAB Abstracts	504,176
S15	TI ((horse n1 human) n3 (relationship* or dyad* or interact* or bond* or communicat*)) OR AB ((horse n1 human) n3 relationship* or dyad* or interact* or bond* or communicat*))	Expanders - Apply equivalent subjects Search modes - Find all my search terms	Interface - EBSCOhost Research Databases Search Screen - Advanced Search Database - CAB Abstracts	81
S14	TI (“Heart rate” OR “heart rate variability” or hydrocortisone) OR AB (“Heart rate” OR “heart rate variability” or hydrocortisone)	Expanders - Apply equivalent subjects Search modes - Find all my search terms	Interface - EBSCOhost Research Databases Search Screen - Advanced Search Database - CAB Abstracts	20,828
S13	TI (Emotional n1 (health OR reaction)) OR AB (Emotional	Expanders - Apply equivalent subjects	Interface - EBSCOhost	673

	n1 (health OR reaction))	Search modes - Find all my search terms	Research Databases Search Screen - Advanced Search Database - CAB Abstracts	
S12	TI (stress* OR fear*) OR AB (stress* OR fear*)	Expanders - Apply equivalent subjects Search modes - Find all my search terms	Interface - EBSCOhost Research Databases Search Screen - Advanced Search Database - CAB Abstracts	366,738
S11	TI (animal N1 (welfare OR well-being OR wellbeing OR wellness)) OR AB (animal N1 (welfare OR well being OR wellbeing OR wellness))	Expanders - Apply equivalent subjects Search modes - Find all my search terms	Interface - EBSCOhost Research Databases Search Screen - Advanced Search Database - CAB Abstracts	13,143
S10	TI ("animal welfare" OR "stress OR "psychological needs" OR "animal behavio#r") OR AB ("animal welfare" OR "stress OR "psychological needs" OR "animal behavio#r")	Expanders - Apply equivalent subjects Search modes - Find all my search terms	Interface - EBSCOhost Research Databases Search Screen - Advanced Search Database - CAB Abstracts	4,450

S9	SU "animal welfare" OR SU "stress" OR SU “stress factors” OR SU "psychological needs" OR SU "animal behaviour" OR SU “basic needs” OR SU “hydrocortisone” OR SU "heart rate"	Expanders - Apply equivalent subjects Search modes - Find all my search terms	Interface - EBSCOhost Research Databases Search Screen - Advanced Search Database - CAB Abstracts	303,431
S8	TI ("horse rider" n1 (relationship* or dyad* or interact* or bond* or communicat*) OR ("horse rider" n1 (relationship* or dyad* or interact* or bond* or communicat*)	Expanders - Apply equivalent subjects Search modes - Find all my search terms	Interface - EBSCOhost Research Databases Search Screen - Advanced Search Database - CAB Abstracts	16
S7	TI (("equine assisted" n1 (therapy or intervention)) OR AB ("equine assisted" n1 (therapy or intervention))	Expanders - Apply equivalent subjects Search modes - Find all my search terms	Interface - EBSCOhost Research Databases Search Screen - Advanced Search Database - CAB Abstracts	22
S6	TI (equin* n2 (activit* or therapy)) OR AB (equin* n2 (activit* or therapy))	Expanders - Apply equivalent subjects Search modes - Find all my search terms	Interface - EBSCOhost Research Databases Search Screen - Advanced Search Database - CAB	609

			Abstracts	
S5	TI ((horse* or equin*) N1 lead*) OR AB ((horse* or equin*) N1 lead*)	Expanders - Apply equivalent subjects Search modes - Find all my search terms	Interface - EBSCOhost Research Databases Search Screen - Advanced Search Database - CAB Abstracts	157
S4	TI ((horse* or equin*) N1 groom*) OR AB ((horse* or equin*) N1 groom*)	Expanders - Apply equivalent subjects Search modes - Find all my search terms	Interface - EBSCOhost Research Databases Search Screen - Advanced Search Database - CAB Abstracts	14
S3	S1 OR S2	Expanders - Apply equivalent subjects Search modes - Find all my search terms	Interface - EBSCOhost Research Databases Search Screen - Advanced Search Database - CAB Abstracts	153,623
S2	SU "horses" OR SU "colts" OR SU "foals" OR SU "mares" OR SU "racehorses" OR SU "stallions"	Expanders - Apply equivalent subjects Search modes - Find all my search terms	Interface - EBSCOhost Research Databases Search Screen - Advanced Search	113,287

			Database - CAB Abstracts	
S1	TI (equin* or horse* or colt* or foal* or mare* or racehorse* or stallion*) OR AB (equin* or horse* or colt* or foal* or mare* or racehorse* or stallion*)	Expanders - Apply equivalent subjects Search modes - Find all my search terms	Interface - EBSCOhost Research Databases Search Screen - Advanced Search Database - CAB Abstracts	138,728
